# DNA metabarcoding analysis revealed a silent prevalence of environmental pathogenic *Leptospira* in urban area of Okinawa Island, Japan

**DOI:** 10.1016/j.onehlt.2025.101016

**Published:** 2025-03-18

**Authors:** Yukuto Sato, Yuiko Hiyajo, Taisei Tengan, Tsurua Yoshida, Yoichiro Uchima, Michinari Tokeshi, Kaori Tsurui-Sato, Claudia Toma

**Affiliations:** a Research Laboratory Center, Faculty of Medicine, University of the Ryukyus, Ginowan, Okinawa, Japan; b Department of Agro-Environmental Sciences, Faculty of Agriculture, University of the Ryukyus, Nishihara, Okinawa, Japan; c The United Graduate School of Agricultural Sciences, Kagoshima University, Kagoshima, Japan; d Department of Bacteriology, Graduate School of Medicine, University of the Ryukyus, Ginowan, Okinawa, Japan

**Keywords:** Environmental DNA, Next-generation sequencing, Eco-epidemiology, One Health, Zoonosis

## Abstract

**Objective:**

Human activities, such as agriculture, environmental manipulation, and city development, have impacted the distribution of flora, fauna, and microbes (including potential human pathogens) at the global level. This study focused on the bacterial genus *Leptospira*, an organism causing leptospirosis that is prevalent in tropical and subtropical regions. We hypothesized that although only a few cases of leptospirosis have been reported in the urban region of main island of Okinawa Prefecture (Okinawa Island, OKI), Japan, *Leptospira* is present in these regions.

**Methods:**

Thirty-four samples were collected from rivers in urban OKI and rural Ishigaki Island (ISG) and analyzed to determine the distribution of *Leptospira* and mammals using environmental DNA (eDNA) metabarcoding. High-throughput sequencing analysis was performed to sequence the polymerase chain reaction products of partial leptospiral 16S rRNA and vertebrate mitochondrial 12S rRNA genes from 16 and 18 river samples of OKI and ISG, respectively, including the waters collected from *Leptospira*-endemic areas in ISG.

**Results:**

*Leptospira noguchii* and *L. interrogans*-related, two *Leptospira* species of the P1+ clade that are pathogenic to humans and mammals, were repeatedly detected in OKI and ISG, supporting our hypothesis. The sequence numbers of the five *Leptospira* species of P1– and P2 clades showed significant correlations with those of cattle (*Bos taurus*) in OKI; however, the potential host animals for P1+ species remain unclear. The total number of leptospiral sequences obtained from the ISG samples was correlated with the distance from the mountainous woodlands.

**Conclusion:**

The pathogenic P1+ *Leptospira* was distributed in urban OKI, in addition to rural ISG. The factors correlated with leptospiral detection, that is, cattle eDNAs and the distance from mountainous forests in OKI and ISG, respectively, suggest the silent prevalence of *Leptospira* in urban and developing regions related to human activities. The findings of the present study provide insights into public health in cities with respect to climate change and possible flood damage.

## Highlights

• Pathogenic *Leptospira* and mammals in Okinawa, Japan, were surveyed using environmental DNA (eDNA) metabarcoding.
• Two pathogenic P1+ *Leptospira* species were detected repeatedly in urban Okinawa and rural Ishigaki Islands.
• *Leptospira* was correlated with cattle and the distance from mountainous forests in Okinawa and Ishigaki, respectively.
• The presence of P1+ *Leptospira* in urban Okinawa should be considered, even though few cases of leptospirosis were reported.

## 1 Introduction

Human activities, such as agriculture, environmental manipulation, city development, and material transportation, have had a significant impact on the terrestrial environment and organismal distribution [1]. These activities have potentially impacted the distribution of zoonotic pathogens. *Leptospira*, a pathogen belonging to the bacterial genus *Leptospira,* causes zoonotic leptospirosis, a disease prevalent in the tropical and subtropical regions. Currently at least 69 species of *Leptospira* have been discovered [[2], [3], [4], [5]] and were classified into 2 subclades of non-pathogenic species (S1 and S2) and 2 subclades of species with variable pathogenic potential (P1 and P2). Within the P1 clade, eight and twelve species were recognized as high- and low-virulence pathogens named as P1+ and P1–, respectively [3,5]. P1+ species, which are highly virulent, are known to infect various mammals and humans [3,5]. Pathogenic *Leptospira* spreads into freshwater environments, such as rivers, ponds, and waterlogged soil, through the urine of infected animals. Humans can contract infection through exposure to contaminated water or soil [4,6], with the majority of individuals contracting leptospirosis through participating in leisure and farming activities [7]. The leptospirosis burden is estimated to be over one million cases, with approximately 60,000 fatalities being reported worldwide each year [8]. Heavy rain and flooding, which are recent effects of climate change, increase the risk of this disease [6,[9], [10], [11], [12], [13]]. The high risk associated with direct exposure to infected animals, such as cattle and rats, has also been emphasized [[14], [15], [16], [17]]. Thus, concerns regarding the spread of leptospirosis through human activities, such as cattle and livestock farming, international trade, and animal invasions, in addition to climate change and floods, have been raised in various countries.

**Fig. 1:**
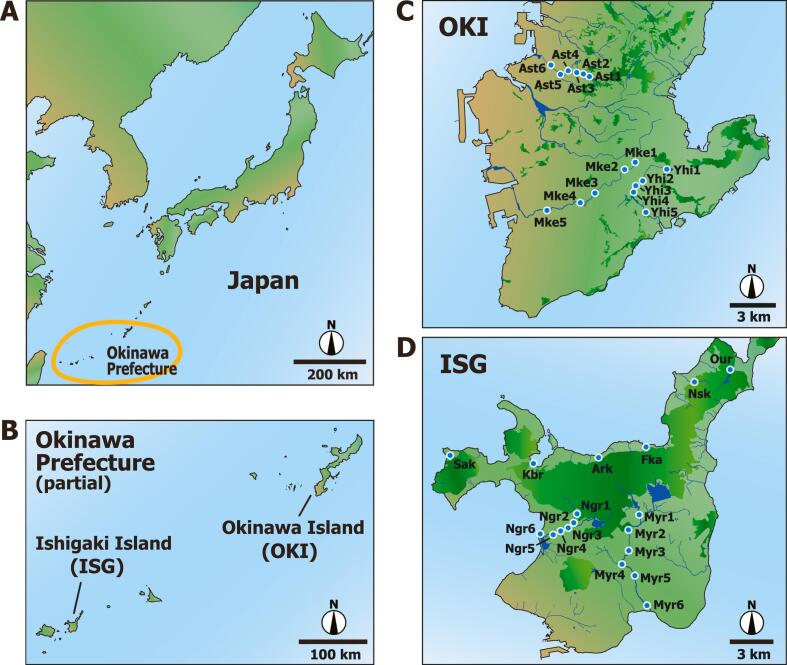
Okinawa (OKI) and Ishigaki (ISG) Islands of the Okinawa Prefecture of Japan and water sampling locations. (A) A map of Japan and the Okinawa Prefecture. (B) A map of OKI and ISG. (C) Sampling locations in the southern area of OKI. The white-edged blue dots indicate the water sampling sites. Yhi, Mke, and Ast indicate the Yuhi, Mukue, and Asato Rivers, respectively. (D) Sampling locations in ISG. Myr and Ngr indicate the Miyara and Nagura Rivers, respectively. Sak, Kbr, Ark, Fka, Nsk, and Our denote Sakieda, Kabira, Arakawa, Fukai, Nosoko, and Oura, respectively. The deep green shading represents mountainous forests and woodlands. The figure was drawn based on map imagery using Adobe Illustrator 2021 v25.2.1. The map imagery was obtained from OpenStreetMap (https://www.openstreetmap.org/) licensed under the Open Data Commons Open Database License by the OpenStreetMap Foundation and the Creative Commons Attribution-ShareAlike 2.0 license (CC BY-SA 2.0). (For interpretation of the references to color in this figure legend, the reader is referred to the web version of this article.)

Approximately half of the cases of leptospirosis in Japan were reported from Okinawa, a Prefecture located in the subtropical area of Japan (Fig. 1A) [18]. Notably, the vast majority of these cases were reported in the northern part of the main island of Okinawa Prefecture (Okinawa Island, OKI) and Yaeyama Islands, which include the Iriomote (IR) and Ishigaki (ISG) (Fig. 1B) [[19], [20], [21]]. The environmental *Leptospira* in IR was analyzed in our previous study [22]. Water-related outdoor activities, such as kayaking, swimming, and farming, have been implicated in the spread of leptospirosis in Okinawa [21,[23], [24], [25]]. In addition, cases of infection occurring among individuals undergoing military training in northern OKI have also been reported [26]. On the other hand, the prevalence of leptospirosis in the southern part of OKI (Fig. 1C), an urban area of the prefecture inhabited by a large portion of the population, is low. This may be attributed to the infection cycle of *Leptospira* among humans, animals, and freshwater remaining incomplete in this area owing to the decreased popularity of river-water activities such as kayaking and swimming. However, *Leptospira* may be also distributed in southern OKI (Fig. 1C). Thus, it is imperative to conduct a systematic survey of the cryptic distribution of *Leptospira* in urban and suburban areas of southern OKI, considering the recent increase in meteorological perturbations, such as typhoons and floods, and the subsequent reports of leptospirosis in Japan [18,27].

Here, we hypothesized that pathogenic *Leptospira* is distributed in the southern part of OKI (Fig. 1C), wherein few cases of leptospirosis have been reported. Environmental DNA (eDNA) metabarcoding [22,[28], [29], [30]], which can analyze leptospiral DNA directly from rivers or terrestrial water bodies such as waterfalls, was conducted to evaluate the prevalence of *Leptospira* in the southern part of OKI. This is a powerful novel technique to analyze marker gene fragments like 16S and 12S rRNA from microbiomes to animals through PCR from the water filtrate materials and DNA sequencing, enabling us to assess biodiversity of targeted pathogens including difficult-to-culture pathogens and to concurrently detect potential host animals [6,28,31]. Here, the *Leptospira* diversity and the ecological aspects were analyzed using the DNA sequences detected in the environmental water samples based on the phylogenetic inference of *Leptospira* species that are difficult to culture or obscure with respect to the serogroups. In addition, eDNA sequencing of vertebrates was performed using the same water samples to enable an exhaustive evaluation of the fauna and animals associated with *Leptospira* [22,[28], [29], [30]]. The environmental *Leptospira* and its candidate host animals in the IR was analyzed in our previous study [22]. Therefore, we aimed to survey and compare representative rivers and waterfalls of southern OKI and ISG (Fig. 1C and D). Water samples collected during the same year and season were subjected to eDNA analysis to detect the environmental *Leptospira*, estimate host animal candidates, and determine their distribution across the two islands. Such a study to associate the pathogen with animal, environmental, and human factors would provide a significant addition to one-health research, offering broader insights into the potential risk factors for leptospirosis in the Okinawa Prefecture, particularly in the southern part of OKI wherein the reports of leptospirosis are rare.

## 2 Materials and methods

### 2.1 Environmental water sampling

A total of 34 environmental water samples were collected from 16 and 18 localities in OKI and ISG, respectively, between December 2023 and January 2024 (Fig. 1; Supplementary material 1). Samples were collected from the Yuhi, Mukue, and Asato Rivers to represent the southern part of the OKI ranging from the western to eastern regions. The annual survey conducted in the prefecture revealed that these rivers exhibited relatively higher values of biochemical oxygen demand (BOD), an indicator of water pollution (maximum 24.0, 6.1, and 3.1 in the Mukue, Yuhi, and Asato Rivers, respectively, from 2013 to 2022). This score was generally lower in other major rivers in OKI like Aja, Kumoji, and Makiminato Rivers (<2.0 during the same year period). Samples were also obtained from the Miyara and Nagura rivers, as well as the Arakawa waterfall in ISG, based on the past outbreaks of leptospirosis according to the prefecture's record. The maximum BOD score was also lower in these rivers in ISG (<2.0 during the same year period). Leisure activities are conducted at these locations. In addition, samples were also collected from small rivers at the Sakieda, Kabira, Fukai, Nosoko, and Oura, to represent the northern area of ISG (Fig. 1).

Surface water samples were obtained mainly from a small bridge with a clean hanging plastic bucket for fishing. Volume in maximum 960 mL of river water (Supplementary material 1) was obtained through on-site filtration with a Sterivex unit (Merck Millipore, Milan, Italy) and a 60-mL disposable syringe (SS-50LZ; Terumo, Tokyo, Japan). The pore size of the Sterivex unit was chosen as 0.45 μm as this size was able to capture *Leptospira* cells in our previous study [28]. Ideally, water sampling must be standardized with regard to the amount (mL) of water; however, given that some rivers are muddier than others, filtration of the same volume of water is difficult to achieve. Therefore, to standardize sampling using an approximate amount of filtered materials, water filtration was conducted until the filtrate clogged the filters. The volume of filtered water (mL) was recorded for each sample. Subsequently, 2.0 mL of DNAiso Reagent (Takara Bio, Shiga, Japan) was added to the Sterivex unit for sample fixation and DNA preservation. A polypropylene Luer-fitting cap (ISIS, Osaka, Japan) was used to achieve tight seal. The samples were stored at room temperature for a duration ranging from a few hours to a few days before being transferred to a University in Okinawa Island. This difference of storage duration in room temperature was not considered to affect the results of eDNA analysis because the DNAiso Reagent fixes DNA and prevents their degradation, as exemplified by detection of diverse *Leptospira* and vertebrate species after more than weeks of room temperature storage shown by our previous study [29]. The samples were stored at a temperature of −25 °C at the University until DNA extraction.

### 2.2 DNA extraction

The DNeasy PowerWater Sterivex Kit (Qiagen, Hilden, Germany) was used in accordance with the standard protocol provided by the manufacturer, with minor modifications, to extract the total eDNA from the water filtrates. In brief, the frozen Sterivex units were placed at room temperature for approximately 15 min before DNA extraction to thaw the DNAiso reagent used for DNA preservation. The reagent was discarded using a disposable syringe (10 mL; Becton, Dickinson and Company). The Sterivex units were incubated at 65 °C on a heat block for 10 min following the addition of the MBL solution at the same temperature to lyse the cells and other biomaterials captured in the filtered residues. Sixty μL of the eDNA solution was obtained from each filter unit through final eDNA elution performed twice using 30 μL of microbial DNA-free water (Qiagen). The concentration (ng/μL) and quality (OD_260/280_) of the eDNA solution was quantified using a Nanodrop 2000c Spectrophotometer (Thermo Fisher Scientific, Waltham, MA, USA), and the solution was stored at −25 °C.

### 2.3 PCR amplification and sequencing for leptospiral and vertebrate metabarcoding

Partial fragments of the 16S rRNA gene of *Leptospira*, which is more specific to pathogenic *Leptospira* than the *lipL32* gene when using environmental samples [29], were amplified from each eDNA sample. The fauna and potential host mammals of *Leptospira* were analyzed using partial fragments of the mitochondrial (mt) 12S rRNA gene of various vertebrates amplified separately from the same sample [22,28,29]. PCR primer sequences targeting leptospiral 16S rRNA [31] and vertebrate mt-12S rRNA (named MiFish primers) [32] were used in addition to the priming site sequences for second-round indexing PCR and DNA sequencing. Furthermore, random hexamer or dimer nucleotides were used to facilitate effective sequencing using the MiSeq platform (Illumina, San Diego, CA, USA) [28,32]. The oligo-nucleotide sequences of the primers have been described in a previous report [22]. The typical target length (base pairs; bp) of PCR primers for leptospiral 16S rRNA and vertebrate mt-12S rRNA were 330 and 169 bp, respectively. The final concentration of each primer for leptospiral and vertebrate rRNAs were 0.47 and 0.30 μM, respectively.

PCR amplification of *Leptospira* and broad bacteria was performed using the Multiplex PCR Assay Kit version 2 (Takara Bio). The 1.5 μL of template eDNA in a total reaction volume of 9.5 μL was subjected to 37 cycles of amplification at an annealing temperature of 50 °C. In addition, three-fifths the concentration of additional broad bacterial 16S rRNA V4 primers (0.28 μM) [33] was used for positive control amplification as described in a previous report [22,30]. The typical target length was 259 bp. Microbial DNA-free water (2.0 μL; Qiagen) was used as the template for negative control PCR. HiFi HotStart ReadyMix (Kapa Biosystems, Woburn, MA, USA) was used to perform PCR of the vertebrates. The 1.5 μL of templates in a total reaction volume of 12.0 μL was subjected to 38 amplification cycles at an annealing temperature of 65 °C. In addition, PrimeSTAR HS DNA polymerase (Takara Bio) was used as a separate PCR for all samples with 1.5 μL of templates in a total reaction volume of 10.0 μL and 35 amplification cycles at an annealing temperature of 55 °C. Negative control PCR was performed for both cycles of PCR using 1.5 μL of microbial DNA-free water (Qiagen) as the template. The use of two DNA polymerases with different fidelities enables the amplification of more diverse DNA types, owing to variations in their susceptibilities to DNA sequences, particularly to secondary structures. The remaining conditions during PCR have been described in a previous report [28,30]. The PCR products obtained through the first round were diluted 50- and 30-fold in leptospiral 16S rRNA and vertebrate mt-12S rRNA, respectively, in microbial DNA-free water (Qiagen). These diluted products were subjected to a second round of PCR for the addition of dual-index tags (D5, D7, A5, and A7 series) and MiSeq flow cell-binding sites (Illumina) using Ex Taq Hot Start Version (Takara) as described previously [28,33].

The tag-indexed PCR products obtained after the second round of PCR were sequenced using MiSeq (Illumina). An equal amount of the PCR products with unique combinations of dual indices for each sample was pooled for semi-quantitative purpose. The pooled samples were purified using 1.0 % L03 agarose gel (Takara Bio) and a MinElute Gel Extraction Kit (Qiagen) in accordance with the standard protocol. AMPure XP magnetic beads (Beckman Coulter, High Wycombe, UK) were used under the standard purification protocol to further purify and concentrate the eluted DNA solution through the removal of short DNA fragments (<100 bp). The sequencing library obtained after purification was quantified using Qubit 3.0 with the dsDNA HS Assay Kit (Thermo Fisher Scientific). The 4 nM library was obtained through dilution with microbial DNA-free water (Qiagen). The volume molarity of the libraries was estimated based on the average molecular weight of a DNA nucleotide (660 g/mol), DNA concentration, and length (bp) of the PCR products after the second round of PCR. The lengths were 526, 454, and 365 bp, respectively, for the leptospiral 16S rRNA, bacterial 16S rRNA V4, and vertebrate mt-12S rRNA genes, respectively. DNA sequencing was performed using the MiSeq Reagent Kit v2 500 cycles (Illumina) for 251 bp paired-end sequencing of leptospiral and bacterial 16S rRNA, and vertebrate mt-12S rRNA genes.

### 2.4 Metabarcoding sequence analysis of Leptospira and vertebrates

The sequence data generated by MiSeq were subjected to quality-based filtering. The software DynamicTrim in the SolexaQA program package was used to remove the 3′-tail nucleotides of each sequence with an error rate of >10^−1^ (Phred score < 10) [34]. The program FLASH [35] was used to merge these paired-end sequences with trimmed tails. Custom Perl scripts [32] were used while filtering to exclude sequences with base call failures (N bases) and those with atypical lengths with respect to the expected PCR target sizes described above. The filter-pass range of the sequence length before primer removal was 180–450 bp for leptospiral 16S rRNA and bacterial 16S rRNA V4. The filter pass range for vertebrate mt-12S rRNA was 204–254 bp. TagCleaner was used to remove primer sequences with a maximum of five base mismatches [36]. Sequences without primers at either end were discarded. A de-replicated sequence was obtained by merging the identical sequences within each sample while adding count information to the sequence name using UCLUST [37]. As a denoising step, singleton sequences for vertebrate mt-12S rRNA in each sample were aligned with ≥2 counts effective sequences at ≥99 % sequence similarity to remove the sequencing error and/or intra-species variation. The number of aligned singletons was added to the count information of the matched effective sequence, and the unmapped ones were discarded. The leptospiral 16S rRNA sequences are genus-specific; consequently, their environmental sequence data, including singletons, were analyzed using molecular phylogeny, as described in the subsection “Molecular phylogenetic analysis.”

The National Center for Biotechnology Information (NCBI) Basic Local Alignment Search Tool (BLAST) Plus program [38] was used to determine the taxonomic origin of the above effective sequences based on the similarity to known reference sequences. The NCBI nucleotide collection database (nt) [39] was used as the reference database for the analysis of the leptospiral 16S rRNA sequences. The NCBI nt and MitoFish database [[40], [41], [42]] were used for the analysis of vertebrate mt-12S rRNA. The sequence similarity and *e*-value threshold in the BLASTN analysis of the leptospiral 16S rRNA were set at 85 % and 10^−3^, respectively. This relatively moderate parameter setting was selected to avoid false negatives (type II errors) in the preliminary sequence annotation. The sequences exhibiting BLAST-hit with <95 bp were discarded. The provisional leptospiral annotations determined according to the BLAST top-hit results were confirmed or corrected based on the molecular phylogenetic analysis with known representative leptospiral 16S rRNA sequences as described in the subsection “Molecular phylogenetic analysis.” The sequence similarity and *e*-value threshold in the BLASTN-based species annotation for vertebrate mt-12S rRNA were set at 90 % and 10^−5^, respectively. The relative strictness of this parameter is attributed to the completeness of the vertebrate mtDNA database, which was generally higher than that of bacteria and *Leptospira*. The sequence counts of the species from the two separate PCRs conducted for vertebrates were summed for each sample.

### 2.5 Molecular phylogenetic analysis

The molecular phylogenetic analysis was conducted for the partial 16S rRNA sequences determined in the eDNA analysis of this study along with the representative reference sequences of the genus *Leptospira* collected based on the phylogenetic trees of Guglielmini et al. [2] and Vincent et al. [3]. The sequences aligned using MAFFT version 7.310 [43] were subjected to molecular phylogeny estimation using the neighbor-joining (NJ) method with MEGA X version 10.2.5 [44]. Kimura's two-parameter model of nucleotide substitution with auto-adjustment of invariable sites and gamma correction parameters was applied. The maximum-likelihood (ML) phylogenetic analysis was also performed for the same data set in the preliminary analysis. The resultant tree was more compatible with the reference leptospiral phylogeny of Guglielmini et al. [2] and Vincent et al. [3] in the NJ method than in ML method, probably because the data set include numbers of closely related sequences with fewer nucleotide substitution. Thus, we adapted the NJ method for the formal analysis. One hundred replications of bootstrapping analysis were conducted to determine the support values for the tree nodes, with nodes with a bootstrap value of ≥50 % being considered reliable. The estimated phylogeny was examined to confirm its compatibility with the reference leptospiral phylogenies reported by Guglielmini et al. [2], Vincent et al. [3], and Giraud-Gatineau et al. [5].

### 2.6 Geographical analysis

The surrounding geography was analyzed to assess the relationship between the detection of *Leptospira* DNA and the geographical characteristics of the sampling locations, considering the possible habitats of mammals based on digital geographic information system (GIS) data. The potential host mammals of *Leptospira,* such as boars, rats, and fruit bats, inhabit mountainous forests and woodlands; therefore, the distances between these mountainous regions and the sampling locations were estimated using two types of geographical indicators: (*i*) the closest summit of the mountain and (*ii*) the closest slope division. The distance (m) from the closest summit of the mountain, which was >180 m above sea level, and the distance (m) from the closest slope division of 20°–30° for each sampling location were determined using QGIS software version 3.32.2 (https://qgis.org), GIS data retrieved from the Geospatial Information Authority of Japan (GSI; https://fgd.gsi.go.jp/download/menu.php), and the global positioning system (GPS) data of each sampling location (Supplementary material 1). The Geographic Information System of Okinawa Prefecture (http://gis.pref.okinawa.jp/pref-okinawa/Portal) was also used. Twenty mountains in the ISG are >180 m above the sea level. On the other hand, there are no mountains >180 m above the sea level in southern OKI owing to its coral reef-based origin with relatively little woodland areas (Fig. 1C and D). Consequently, OKI was not included in this geographic analysis.

### 2.7 Statistical analysis

The Welch's *t-*test based on mean and standard errors were used to assess the significance of the difference in the average values. This test was applied to the amount of water samples filtered from each location (mL) and the concentration (ng/μL) of the extracted eDNA from these samples. The Mann–Whitney *U* test was used to assess the significance of the difference in the number of eDNA sequences of the *Leptospira* between the islands. Two generalized linear mixed models (GLMMs) with quasi-Poisson or negative binomial regression were constructed to examine the effects of distance from the closest mountains (Supplementary material 2) on eDNA detection of *Leptospira*. These regressions assumed to model count data comprising non-negative integers without an upper limit, such as numbers of DNA sequences. The GLMM analysis estimated parameters, such as the exponential coefficient, exponential standard error, *z* score, and Akaike's information criterion (AIC), using the maximum likelihood method to predict a response variable by fitting data to the models. The ratio of the total number of genus *Leptospira* sequences per number of raw sequences was defined as the response variable for each model, which included the shortest distance from the mountainous area as the fixed effect, the sampling location as a random effect, and number of raw sequences from each sample as an offset. Pearson's product-moment correlation coefficient (*r*) was used to assess the relationship between the number of eDNA sequences detected from *Leptospira* and vertebrates. The Benjamini–Hochberg method was used to assess the false discovery rate (FDR) of *r*. All statistical analyses were conducted using *R* version 4.3.3 (https://www.R-project.org) [45].

### 2.8 Data availability statement

The raw sequence reads generated using MiSeq in the current study are available in the DDBJ Sequence Read Archive (DRA; under BioProject accession numbers PRJDB18960 and PRJDB18961 for leptospiral and vertebrate metabarcoding analyses, respectively). All relevant data are available in the manuscript and Supporting materials.

## 3 Results

### 3.1 Environmental DNA metabarcoding detection of Leptospira

In total 34 samples were collected from rivers and freshwater areas to determine the distribution pattern of *Leptospira* species in OKI and ISG in the subtropical Okinawa Prefecture, Japan (Fig. 1; Supplementary material 1), with the mean (± S.E.) volume of filtered water of 367.7 ± 38.5 mL. The mean total eDNA extracted from each sample was 9.05 ± 1.42 ng/μL, with a mean OD_260/280_ quality of 1.74 ± 0.03. The average amount of filtered water and the concentration of the extracted eDNA did not differ significantly between OKI and ISG (383.8 ± 45.8 mL and 353.3 ± 61.3 mL in OKI and ISG, respectively: Welch's *t-*test, *p* = 0.694; 10.21 ± 2.42 ng/μL and 8.02 ± 1.63 ng/μL in OKI and ISG, respectively: Welch's *t-*test, *p* = 0.459). Similarly, no significant differences were observed in these values between the samples collected on sunny/cloudy and rainy days (362.7 ± 47.4 mL and 383.8 ± 59.2 mL in the sunny/cloudy and rain samples, respectively: Welch's *t-*test, *p* = 0.785; 9.46 ± 1.81 ng/μL and 7.73 ± 1.44 ng/μL in the sunny/cloudy and rain samples, respectively: Welch's *t-*test, *p* = 0.461). The mean OD_260/280_ quality score was significantly higher in OKI than in ISG (1.84 ± 0.02 and 1.64 ± 0.02 in OKI and ISG, respectively: Welch's *t-*test, *p* < 0.001).

Partial fragments of the leptospiral and bacterial 16S rRNA gene were amplified from each eDNA sample in a multiplex manner and sequenced by MiSeq. A total of 1,037,738 pairs of raw sequences were obtained, with an average of 30,522 ± 803 reads per sample (a read means a pair of raw DNA sequence of forward and reverse direction). In total 1,007,225 reads, with 29,624 ± 830 reads per sample, were retained as quality-filtered sequences after primary data filtering. Each primer end of the amplified bacterial 16S rRNA V4 region were detected in 999,747 reads (29,404 ± 844 reads per sample), whereas those of leptospiral 16S rRNA were detected in 7478 reads (220 ± 73 reads per sample).

**Fig. 2:**
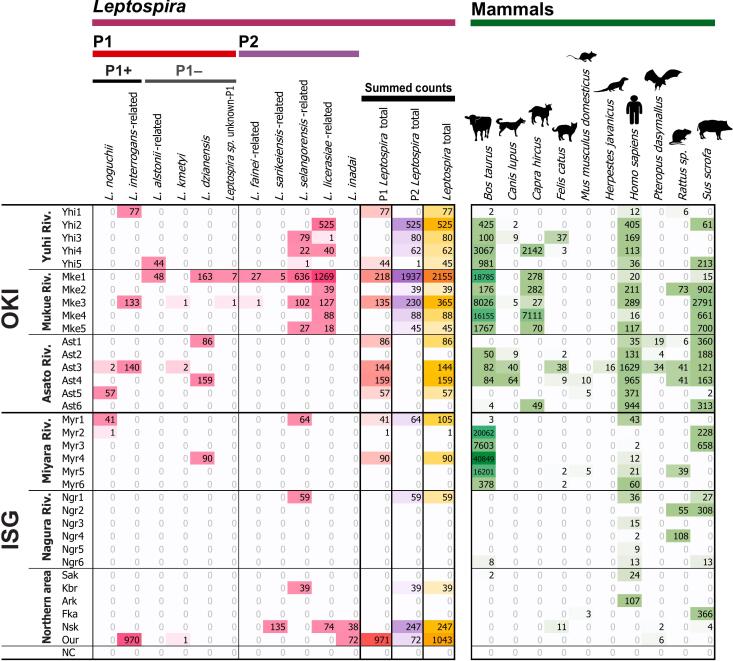
Environmental DNA detection of partial leptospiral 16S rRNA and mammal mitochondrial 12S rRNA genes. The columns denote the operational taxonomic unit (OTU) of *Leptospira* and species of mammals detected through environmental DNA (eDNA) metabarcoding. The rows indicate the sample names and number of eDNA sequences detected in each sample. The sequence numbers are shown, with the colored matrices in magenta and green shading representing *Leptospira* and mammals, respectively. The color intensity is relative to the sequence numbers. P1 and P2 denote the phylogenetic subclades of pathogenic *Leptospira* based on the definitions of Vincent et al. [3] and those of the present molecular phylogenetic analysis (Supplementary material 3). P1+ and P1– indicate the species categories of the pathogenic P1 *Leptospira* based on the definitions of Giraud-Gatineau et al. [5]. The “P1 *Leptospira* total,” “P2 *Leptospira* total,” and “*Leptospira* total” columns indicate the summed sequence numbers across P1, P2, and all *Leptospira* OTUs, respectively. These categories are depicted as colored matrixes with red, purple, and brown shading, respectively. NC indicates a PCR negative control sample (RNase-free water). (For interpretation of the references to color in this figure legend, the reader is referred to the web version of this article.) Environmental DNA detection of partial leptospiral 16S rRNA and mammal mitochondrial 12S rRNA genes. The columns denote the operational taxonomic unit (OTU) of *Leptospira* and species of mammals detected through environmental DNA (eDNA) metabarcoding. The rows indicate the sample names and number of eDNA sequences detected in each sample. The sequence numbers are shown, with the colored matrices in magenta and green shading representing *Leptospira* and mammals, respectively. The color intensity is relative to the sequence numbers. P1 and P2 denote the phylogenetic subclades of pathogenic *Leptospira* based on the definitions of Vincent et al. [3] and those of the present molecular phylogenetic analysis (Supplementary material 3). P1+ and P1– indicate the species categories of the pathogenic P1 *Leptospira* based on the definitions of Giraud-Gatineau et al. [5]. The “P1 *Leptospira* total,” “P2 *Leptospira* total,” and “*Leptospira* total” columns indicate the summed sequence numbers across P1, P2, and all *Leptospira* OTUs, respectively. These categories are depicted as colored matrixes with red, purple, and brown shading, respectively. NC indicates a PCR negative control sample (RNase-free water). (For interpretation of the references to color in this figure legend, the reader is referred to the web version of this article.)

A total of 5511 leptospiral sequences were identified from the above 7478 16S rRNA gene sequences (pink, red, purple, and yellow-brown shading in Fig. 2). Among the 7478 sequences, 28 were discarded owing to a shorter BLAST-hit of <95 bp, 56 were discarded as they exhibited a BLAST-hit to bacteria other than *Leptospira*, and 1883 were discarded as they exhibited no BLAST hits against the NCBI nt database [32]. The leptospiral annotation of the remaining 5 511 sequences, which was primarily achieved from the BLAST top-hit against the NCBI nt, was carefully examined and corrected based on the results of molecular phylogenetic analysis with the reference 16S rRNA gene sequences of known *Leptospira* species (Supplementary material 3), obtaining phylogenetically defined environmental *Leptospira* sequences from OKI and ISG.

### 3.2 Detection, distribution and species diversity of Leptospira in OKI and ISG

Fig. 2 presents the operational taxonomic units (OTUs) and number of sequences of *Leptospira* detected from each location. Eleven OTUs of *Leptospira* were identified through species annotation based on the molecular phylogenetic tree (Supplementary material 3). The leptospiral eDNA included sequences genetically related to *L. noguchii* and *L. interrogans* of the P1+ species; *L. alstonii*, *L. kmetyi*, and *L. dzianensis* of the P1– species from the P1 subclade, and *L. fainei*, *L. sarikeiensis*, *L. selangorensis*, *L. licerasiae*, and *L. inadai* from the P2 subclade [2,3,5]. The *Leptospira* clade provisionally named *Leptospira* sp. unknown-P1 (Fig. 2) did not include known closely-related *Leptospira* from the P1 subclade (Supplementary material 3). The species annotation obtained was partially obscure and limited in terms of resolution owing to the shorter eDNA-derived sequences; however, the classification of the P1 and P2 clades and their respective total number of sequences were not affected.

Clade P1+ *Leptospira* was detected in both OKI and ISG (Fig. 2). Notably, compared with that in ISG, relatively larger numbers of *Leptospira* OTUs and sequences were detected in OKI. Ten and eight *Leptospira* OTUs were identified in OKI and ISG, respectively. The Shannon's α index for species diversity of *Leptospira* based on the number of sequences was, on average, 0.28 ± 0.11 and 0.27 ± 0.15 in OKI and ISG, respectively, indicating no statistically significant difference (Mann–Whitney *U* test, *p* = 0.904). The total number of *Leptospira* sequences detected in OKI was higher than that detected in ISG (in average 245.4 ± 131.9 and 88.0 ± 58.1 reads, respectively), and this difference was statistically significant (Mann–Whitney *U* test, *p* = 0.009). Division of the P1 and P2 clades of *Leptospira* revealed a marginal difference: 57.5 ± 18.0 and 61.3 ± 53.8 for P1 in OKI and ISG, respectively (Mann–Whitney *U* test, *p* = 0.073); 187.9 ± 121.4 and 26.7 ± 14.3 for P2 in OKI and ISG, respectively (Mann–Whitney *U* test, *p* = 0.082). Several OTUs (7/11, 64 %) were identified on both islands; however, *L. alstonii*-related, *L. fainei*-related, and *Leptospira* sp. unknown-P1 were identified only in OKI, whereas *L. inadai* was identified only in ISG (Fig. 2).

### 3.3 Environmental DNA metabarcoding detection of vertebrates and its correlation with Leptospira

Partial fragments of vertebrate mt-12S rRNA gene from each eDNA sample were amplified and sequenced by MiSeq, obtaining 1,162,707 pairs of raw sequences with an average of 17,099 ± 980 reads per one of the two PCRs with different DNA polymerases from each sample. A total of 821,496 reads were retained as quality-filtered sequences after primary data filtering, with 12,081 ± 1029 reads per PCR with each primer end of the amplified vertebrate mt-12S rRNA identified.

These putative vertebrate sequences yielded 799,319 effective vertebrate sequences (green shading in Fig. 2). Among the filtered 821,496 sequences, 803,132 non-singleton (≥2 counts) effective sequences were conferred species annotation after re-mapping analysis of the singleton sequences and BLAST-based analysis using a customized version of the MiFish pipeline [[40], [41], [42]] with tetrapod data from the NCBI nt database [39]. These sequences were annotated as follows: mammalian, 159,376; non-mammalian tetrapods (avian and reptile), 1834; and teleost fish, 638,109. The remaining 3813 sequences exhibited no BLAST hits. The annotated vertebrate eDNA sequences denoted 157 vertebrate species, comprising 10 mammalian, 12 avian, two reptilian, and 133 teleost fish species. Fig. 2 (right-hand panel) presents the mammalian species, which are potential hosts for *Leptospira*, and their number of sequences detected from each sample. Supplementary material 4 lists the vertebrate species and their sequence counts.

**Fig. 3:**
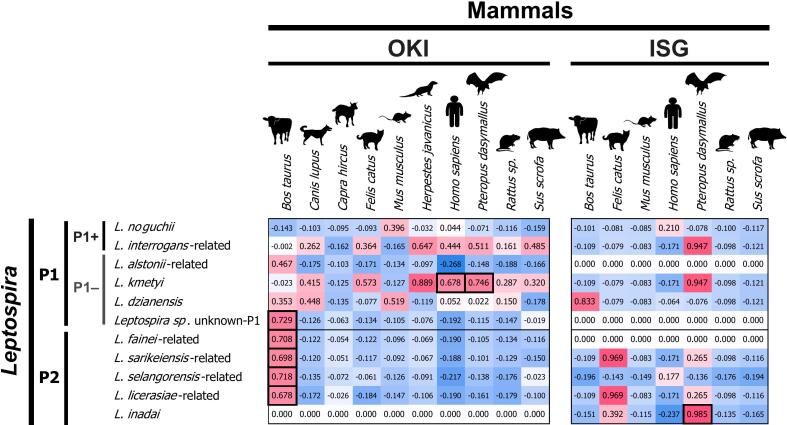
Correlation between eDNA detection of *Leptospira* and mammals. Pearson's correlation coefficients between the detected numbers of sequences of *Leptospira* (results from leptospiral 16S rRNA) and those of the mammals (results from mitochondrial 12S rRNA) are indicated in red (positive value) to blue (negative value) shading. The bold boxes indicate significant positive correlation between the *Leptospira* OTU and mammalian species, wherein either species were detected repeatedly at least two times from respective Islands, after Benjamini–Hochberg correction of false discovery rate at *q* < 0.01. P1 and P2 denote phylogenetic subclades of pathogenic *Leptospira* based on the definitions of Vincent et al. [3] and present molecular phylogenetic analysis (Supplementary material 3). P1+ and P1– indicate the species categories of the pathogenic P1 *Leptospira* based on the definitions of Giraud-Gatineau et al. 2024 [5]. (For interpretation of the references to color in this figure legend, the reader is referred to the web version of this article.) Correlation between eDNA detection of *Leptospira* and mammals. Pearson's correlation coefficients between the detected numbers of sequences of *Leptospira* (results from leptospiral 16S rRNA) and those of the mammals (results from mitochondrial 12S rRNA) are indicated in red (positive value) to blue (negative value) shading. The bold boxes indicate significant positive correlation between the *Leptospira* OTU and mammalian species, wherein either species were detected repeatedly at least two times from respective Islands, after Benjamini–Hochberg correction of false discovery rate at *q* < 0.01. P1 and P2 denote phylogenetic subclades of pathogenic *Leptospira* based on the definitions of Vincent et al. [3] and present molecular phylogenetic analysis (Supplementary material 3). P1+ and P1– indicate the species categories of the pathogenic P1 *Leptospira* based on the definitions of Giraud-Gatineau et al. 2024 [5]. (For interpretation of the references to color in this figure legend, the reader is referred to the web version of this article.)

A correlation between eDNA detection of *Leptospira* and mammals was observed in clade P2 *Leptospira* and the cattle in OKI. Fig. 3 depicts the Pearson's correlation coefficient *r* scores. The bold box indicates statistically significant results, i.e., both *Leptospira* and vertebrates were detected more than twice in these samples. Significant co-occurrence was observed in the cattle *Bos taurus,* with five OTUs of *Leptospira* in OKI: *L. fainei*-, *L. sarikeiensis*-, *L. selangorensis*-, and *L. licerasiae*-related of the P2 clade and *Leptospira* sp. unknown-P1 species of the P1– clade (*r* = 0.678 to 0.729, *d.f*. = 14, *p* = 0.001 to 0.004; Benjamini–Hochberg [BH]-corrected FDR *q* < 0.01). In addition, *L. kmetyi* showed significant correlation with humans and the Ryukyu fruit bat *Pteropus dasymallus* in OKI (*r* = 0.678, *d.f*. = 14, *p* = 0.004 and *r* = 0.746, *d.f*. = 14, *p* < 0.001 for humans and bats, respectively; BH-corrected FDR *q* < 0.01). The *L. inadai* showed a significant correlation with the Ryukyu fruit bat *P. dasymallus* in ISG (*r* = 0.985, *d.f*. = 16, *p* < 0.001; BH-corrected FDR *q* < 0.01).

**Fig. 4:**
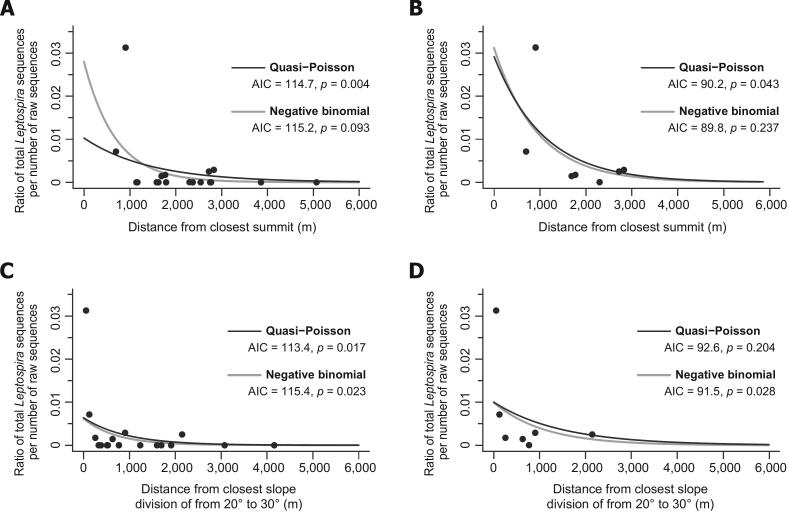
Association between environmental *Leptospira* detection and distances from the closest mountainous area in the Ishigaki Island (ISG). The vertical axis indicates the ratio of the total number of the sequences of *Leptospira* per number of raw sequences after quality filtering from each sampling location. The horizontal axis denotes the distance (m) from the closest mountainous area of each sampling location estimated using two indicators: (A) (B) distance (m) from the closest summit of mountains >180 m above sea level; (C) (D) distance (m) from the closest slope division of 20–30°. Non-linear regression lines of quasi-Poisson and negative binomial models are represented by black and gray lines, respectively. AIC indicates Akaike's information criterion score, whereas *p* indicates the *p*-value of the slope coefficient for the distance parameter. All sampling locations of ISG were analyzed in the results shown in panels A and C. Sampling locations with no leptospiral detection (0 sequences) were excluded from the analysis shown in panels B and D.

### 3.4 Correlation between eDNA detection of Leptospira and the geographic factors

A significant association was observed between the total number of *Leptospira* sequences detected (Fig. 2) and distance from the closest mountainous area of each sampling location in ISG (Fig. 4; Supplementary material 2). Nonlinear regression analysis based on quasi-Poisson or negative binomial distribution yielded two lines, indicating that at least one of the slope coefficients was significant based on the distance from the closest summit or the closest slope division of 20–30°, showing higher detection rate of *Leptospira* in the island near the mountainous area. The AIC, exponential coefficient, *z* value, and *p*-value of the regression using all sampling point data were 114.7, 0.999, −2.864, and 0.004 and 115.2, 0.999, −1.679, and 0.093 for quasi-Poisson and negative binomial regressions, respectively, when distance measures focusing on the summit of mountains were used (Fig. 4A). The AIC, exponential coefficient, *z* value, and *p*-value were 90.2, 0.999, −2.024, and 0.043 and 89.8, 0.999, −1.183, and 0.237 for quasi-Poisson and negative binomial regressions, respectively, using sampling points with more than one sequence of *Leptospira* (Fig. 4B). The AIC, exponential coefficient, *z* value, and *p*-value of the regression using all sampling point data were 113.4, 0.999, −2.387, and 0.017 and 115.4, 0.999, −2.277, and 0.023 for quasi-Poisson and negative binomial regressions, respectively, when distance measures focusing on the slope division from 20 to 30° were used (Fig. 4C). The AIC, exponential coefficient, *z* value, and *p*-value were 92.6, 0.999, −1.270, and 0.204 and 91.5, 0.999, −2.192, and 0.028 for quasi-Poisson and negative binomial regressions, respectively, using sampling points with more than one sequence of *Leptospira* (Fig. 4D). Lower *p*-values were observed when data from all sampling points were used (*p*-values from 0.004 to 0.093; Fig. 4A and C), whereas lower AICs values were observed when data from sampling points with >1 sequence of *Leptospira* were used (AIC scores from 89.8 to 92.6) (Fig. 4B and D).

## 4 Discussion

The present eDNA analysis of *Leptospira* conducted using environmental water samples acquired from OKI and ISG revealed the presence of pathogenic P1+ clade *Leptospira* (*L. noguchii* and *L. interrogans*-related) in OKI and ISG, supporting the hypothesis that *Leptospira* is also distributed in urban OKI (Fig. 2). In Japan, leptospirosis is considered a notifiable disease under the Infectious Diseases Control Law since November 2003. In Okinawa Prefecture, most of the cases were reported in the northern part of Okinawa and Yaeyama region every year [25], whereas clinical cases were reported in southern OKI sporadically. We consider a silent *Leptospira* prevalence in the southern urban OKI, because only one clinical case was reported in Okinawa Prefectural report on 2023 despite the presence of pathogenic P1+ species in the environment. The finding that more abundant and diverse *Leptospira* sequences and OTUs were detected in OKI than in ISG, where water-related recreational activities are the main source of infection, highlighted the importance of human behavioral factors increasing the risk of infection.

The number of species and Shannon's α index of the *Leptospira* diversity in OKI (10 and 0.28, respectively) were comparable with or higher than those detected in ISG (8 and 0.27, respectively). In addition, the P1– OTUs related to *L. alstonii* and *Leptospira* sp. unknown-P1 were only detected in OKI. The present study was conducted in the same season using identical methods of the experiments in OKI and ISG. Thus, the differences in sequence abundance and diversity of *Leptospira* detected in the present study can be considered to reflect the actual biomass on these two islands. Although the water pollution indicator BOD was generally higher in the sampling rivers of OKI than in those of ISG, there were no significant differences in the average amount of filtered water and the concentration of extracted eDNA between OKI and ISG, implying that our standardization method of water sampling based on amounts of filtered materials worked to some extent. However, the higher OD_260_ of eDNA of OKI may have partially affected the sequence abundance of the *Leptospira* in OKI. *Leptospira* including P1+ pathogenic ones existed in the southern part of OKI, in addition to the known *Leptospira*-endemic region of ISG, although few incidences of leptospirosis in urban OKI have been reported. The spread of leptospirosis among humans can be generally traced back to water activities, such as leisure activities in rivers or farming [17,21,[23], [24], [25]]. The already-reported *Leptospira*-endemic area and the actual area of distribution of *Leptospira* may not match completely. Our findings showing a silent prevalence of *Leptospira* in urban areas such as southern OKI, where contact between humans and *Leptospira* is usually limited, should be considered when climate factors such as typhoon and heavy rains with flooding can increase the human risk of infection [30] by contaminated environmental water in the urban regions of OKI. The integration of regional or global climate datasets into the statistical analysis of eDNA data would be important in future studies.

Detection of cattle eDNA and the distance from mountainous areas in OKI and ISG were identified as the factors partly correlated with the detection of *Leptospira*. Natural reservoirs of *Leptospira* include various species of wild mammals, such as rats and boars. No mammalian species that exhibited particular correlations with *Leptospira* detection was observed in rural ISG (Fig. 3). This may indicate that the source of *Leptospira* is the various mammals that inhabit the mountainous forests or soil [46] in ISG, not any particular mammalian species such as livestock. This assumption is supported by the finding of the present study that the intensity of *Leptospira* eDNA detection is largely associated with the distance from mountainous areas inhabited by wild mammals (Fig. 4). On the other hand, the cattle in OKI have been implied as one of the hosts for *Leptospira* (Fig. 3); however, this association is limited to a part of the P1– and P2 clade *Leptospira* that may not be the main pathogens affecting humans [5]. A relative increase in cattle farming has been observed in OKI since the 1980s, leading to an increase in the amount of cattle-related sewage flow into local rivers. Piled cattle feces frequently seen in farming fields in southern OKI can be the source of river and environmental *Leptospira* and other bacteria streamed out by rain fall. Thus, although *Leptospira* eDNAs were detected in both OKI and ISG as environmental bacteria, a remarkable difference may exist between these regions in terms of the natural source and infection cycle of *Leptospira*. The association between *Leptospira* and non-native cattle observed in OKI (Fig. 3) suggests that relatively recent human activities, such as livestock farming, may have at least partially led to an increase in the prevalence of *Leptospira* [[14], [15], [16]] in these urban areas.

Findings related to the presence of *Leptospira* have been reported in peri-domestic areas of various countries: *L. interrogans* from animals such as skunks and raccoons near the city areas of Los Angeles, USA [47]; *L. interrogans* and *L.* *borgpetersenii* from dogs in the parks of Sydney, Australia [48,49]; *L. interrogans* and *L. kirschneri* from rodents and shrews in the city areas of Lyon, France [50]; *L. interrogans* from peri-domestic water sources of Iquitos, Peru [51]; *L. interrogans* and *L. borgpetersenii* correlated with rodent detection in the Los Rios Region, Chile [31]; *L. interrogans* and *L. borgpetersenii* associated with cattle and water buffalo eDNA in Kandy, Sri Lanka [29]; and other similar examples from Uruguay [52], Brazil [53], Philippines, and Japan [54]. A severe case of leptospirosis was recently reported in a man in New England, USA. The source of infection was estimated to be a pet dog that contracted leptospirosis from a small river in a city park [55]. The prevalence of pathogenic P1+ *Leptospira* in cities can potentially lead to an outbreak following heavy rain and flooding related to global climate change [6,[9], [10], [11], [12], [13],^27^,^28^,^46^]. Leptospirosis has been reported more frequently during the summer season in OKI; this may be attributed to the occurrence of typhoons during this season [18]. Reports of outbreaks of leptospirosis after typhoons in other regions of Japan have also increased in recent years [18,27]. The emerging threat of leptospirosis, that is, the increase in human contact with carriers of *Leptospira* and soils in city regions, the urbanization of *Leptospira* distribution owing to human activities such as livestock farming, and an increase in the incidence of flood disasters [4,30,56], should be heeded to in various countries and regions, including developed countries.

The present study has some limitations. The taxonomic resolution of the leptospiral eDNA analysis was not sufficient at the species level (Supplementary material 3) owing to the shorter length of the 16S rRNA gene amplification. The major phylogenetic clades of *Leptospira* P1 and P2 [2,3] could be distinguished; however, the relationship between closely-related *Leptospira* species has not been fully resolved. The P1+ (high-virulence pathogens) and P1– (low-virulence pathogens) groups, the phylogenetic groups within subclade P1 [3,5], exhibited polyphyletic relationships in the present phylogenetic tree, which may be attributed to the lack of intra-subclade resolution. In addition, the reference sequence of P1– *L. gomenensis* was clustered with that of P1+ *L. interrogans*, exhibiting limited resolution at the species level (Supplementary material 3). A relationship among the saprophytic subclades of *Leptospira* (S1 and S2) was also not resolved. Additional marker genes, such as *lfb1* [57], *secY* [58], and *flaB* [54], may have to be incorporated to improve the resolution and accuracy of *Leptospira* metabarcoding. In addition, the results of the present quantitative analysis of the number of eDNA sequences must be interpreted with caution. The amount of water filtered during sampling (Supplementary material 1) varied; this may have affected the diversity of *Leptospira* and animal species detected. As we tried to standardize our eDNA sampling among rivers based on the amounts of filtered materials, this method may be further improved by taking parameters like water turbidity to statistically evaluate and correct the eDNA sequence abundance. The genomic copy number of the 16S rRNA gene varies across bacterial species, ranging from one to about 15 [59]. The copy number of the mitochondrial DNA used as a taxonomic marker of vertebrates also varies across animal species, tissues, and cells [60]. Thus, our results based on the number of eDNA sequences have technical limitations, although the number was thought to be largely proportional to the biomass. The eDNA detection of *Leptospira* only reinforces its presence, and the correlation between *Leptospira* and animals only indicates possible associations between them. Direct isolation and culturing of *Leptospira* from the environment and animals may provide substantial evidence [22].

In summary, the present study demonstrated the distribution of P1+ pathogenic *Leptospira* species, that is, *L. noguchii* and *L. interrogans*-related OTUs, in the urban regions of OKI through eDNA metabarcoding analysis. While few cases of leptospirosis have been reported in this region, such a prevalence of pathogenic *Leptospira* in urban or peri-domestic regions has been reported in various countries, including the USA, France, and Brazil, which may be attributed to rodents, dogs, cattle, raccoons, and wastewater. In addition, in the present study, the association of cattle eDNA was exhibited by five of the ten *Leptospira* species detected in southern OKI, implying that the prevalence of *Leptospira* in urban areas may be related to human activities, such as livestock farming. The findings of this study provide important insights into the potential risk of leptospirosis transmission in the urban areas of Japan and developed countries. They also provide useful information on public health and livestock management with respect to possible climate change and flood disasters.

The following are the supplementary data related to this article.

Supplementary material 1 Sample names and information, locations, weather, sampling dates, and concentration and quality of the extracted DNA. Supplementary material 1

Supplementary material 2 Distance data from the geographic analysis of the sampling sites on Ishigaki Island. Supplementary material 2

Supplementary material 3 Molecular phylogenetic tree of the partial leptospiral 16S rRNA sequences detected from freshwater eDNA samples and representative *Leptospira* species. The operational taxonomic units (OTUs) indicated in black letters represent the partial 16S rRNA sequences determined from the 34 eDNA samples acquired from Okinawa and the Ishigaki Islands (ranging from 293 to 294 bp). The sample names and total sequence counts are denoted within the sequence names. The sequence counts are indicated after the small letter “s” at the end of sequence names. The OTUs indicated in red, green, and blue letters represent 16S rRNA genes of representative *Leptospira* species obtained from the National Center for Biotechnology Information (NCBI) nucleotide database. The GenBank accession numbers of these reference sequences are presented in the sequence names. The subclade annotations P1, P2, P1+, P1-, S1, and S2 are based on the definitions of the studies by Vincent et al. 2019 [3] and Giraud-Gatineau et al. 2024 [5], where P and S indicate pathogenic and saprophytic clades of *Leptospira*. In total, 226 nucleotide sites in 164 sequences were aligned and analyzed. The values on the tree nodes denote the percentage of support for the node estimated from 100 bootstrap replications. Supplementary material 3

Supplementary material 4 Sequence counts and taxonomic profiling of the vertebrates detected in water samples. Supplementary material 4

## CRediT authorship contribution statement

**Yukuto Sato:** Writing – review & editing, Writing – original draft, Visualization, Validation, Supervision, Software, Resources, Project administration, Methodology, Investigation, Funding acquisition, Formal analysis, Data curation, Conceptualization. **Yuiko Hiyajo:** Methodology, Investigation, Formal analysis, Data curation, Conceptualization. **Taisei Tengan:** Investigation, Formal analysis, Data curation, Conceptualization. **Tsurua Yoshida:** Methodology, Investigation, Data curation, Conceptualization. **Yoichiro Uchima:** Investigation, Data curation, Conceptualization. **Michinari Tokeshi:** Investigation, Data curation, Conceptualization. **Kaori Tsurui-Sato:** Writing – review & editing, Writing – original draft, Visualization, Validation, Methodology, Investigation, Formal analysis, Data curation, Funding acquisition. **Claudia Toma:** Writing – review & editing, Validation, Methodology, Data curation, Conceptualization.

## Ethics approval and consent to participate

Not applicable.

## Declaration of Generative AI and AI-assisted technologies in the writing process

The authors declare that they did not use any generative AI or AI-assisted technologies during the writing process.

## Funding

This study was funded by the Japan Society for the Promotion of Science (JSPS) Grants-in-Aid for Scientific Research - Grant ID: 20K12258 and 23K28279 to Y.S, and 24K03131 to K.T.S.

## Declaration of competing interest

On behalf of all authors, the corresponding author states that there are no competing interests to declare.

## Data Availability

Raw sequence reads generated by MiSeq in the current study are available in the DDBJ Sequence Read Archive (DRA) for leptospiral and vertebrate metabarcoding data. BioProject accession numbers: PRJDB18960 and PRJDB18961.
