# Supplementary figures and images for: DNA metabarcoding analysis revealed a silent prevalence of environmental pathogenic Leptospira in urban area of Okinawa Island, Japan
Source: One Health. 2025 Mar 18;20:101016. doi: 10.1016/j.onehlt.2025.101016 (PMC11987687; doi:10.1016/j.onehlt.2025.101016)

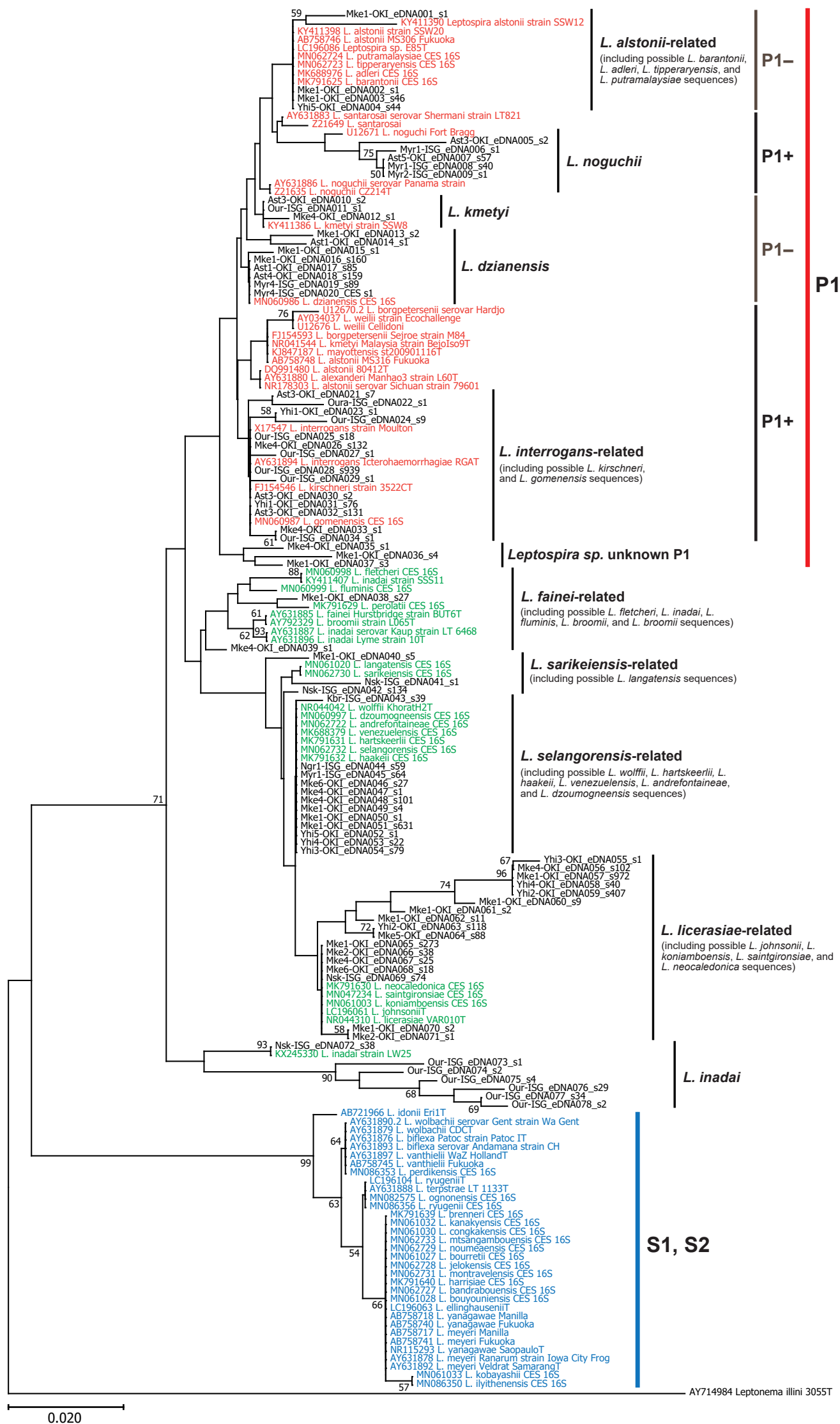

Supplement: Supplementary material 3 — Molecular phylogenetic tree of the partial leptospiral 16S rRNA sequences detected from freshwater eDNA samples and representative Leptospira species. The operational taxonomic units (OTUs) indicated in black letters represent the partial 16S rRNA sequences determined from the 34 eDNA samples acquired from Okinawa and the Ishigaki Islands (ranging from 293 to 294 bp). The sample names and total sequence counts are denoted within the sequence names. The sequence counts are indicated after the small letter “s” at the end of sequence names. The OTUs indicated in red, green, and blue letters represent 16S rRNA genes of representative Leptospira species obtained from the National Center for Biotechnology Information (NCBI) nucleotide database. The GenBank accession numbers of these reference sequences are presented in the sequence names. The subclade annotations P1, P2, P1+, P1-, S1, and S2 are based on the definitions of the studies by Vincent et al. 2019 [3] and Giraud-Gatineau et al. 2024 [5], where P and S indicate pathogenic and saprophytic clades of Leptospira. In total, 226 nucleotide sites in 164 sequences were aligned and analyzed. The values on the tree nodes denote the percentage of support for the node estimated from 100 bootstrap replications. [file mmc3.pdf]
